# Nasal Obstruction—III

**Published:** 1908-08-01

**Authors:** 


### Laryngology and Rhinology.

### NASAL OBSTRUCTION—III.

Deflection of the septum nasi is a very fertile source of obstruction; indeed, very few adult septa are completely straight, though deflection is far less common in children under the age of seven. The causation is often obscure; many are the result of traumatism, often of a slight forgotten injury in early life. Others appear to be due to some defect of growth, and the highly arched palate so often associated with adenoids may possibly cause a deflection by allowing insufficient room for expansion.

The deflection is usually most marked in front in the region of the triangular cartilage; it may assume a great variety of forms, sharp-edged or rounded, a simple or an S-shaped curve, from before backwards or from above downwards. Two types, which may be combined, are prominent. In the one the deflection runs backwards and upwards along the upper border of the vomer at its junction, in front with the triangular cartilage, and behind with the perpendicular plate of the ethmoid; a ridge is thus formed projecting into one naris with a slight concavity on the opposite side, or there may be a ridge formed by the cartilage on the one side and on the other, at a slightly lower level, one formed by the bony vomer. This ridge often has a sharp edge, and seen foreshortened by rhinoscopy, was formerly described as a "nasal spur"; more rarely the projection is very short, and may properly be called a spur. Where this ridge meets the floor of the nose in front, the bone formed by the crest of the superior maxillæ, is often thickened and causes a large part of the obstruction. In the second type, the deflection is about a more vertical line running upwards and slightly forwards at nearly a right angle to the vomerine ridge; the angle thus formed may be rounded or sharp; it involves the triangular cartilage, and is usually found well forward within half an inch of the nostril.

Dislocation of the anterior end of the triangular cartilage is associated with deflection of the septum, and the dislocated end, like the edge of a cup, projects into the nostril on the side of the concavity. It is displaced from its seat between the two lower lateral cartilages of the nose, and forms a smooth, firm forwardly-projecting ridge just behind the movable parts of the columella. Dislocation of the inner limb of the lower lateral cartilage is another cause of obstruction; the projection is more movable and lies further forward on the inner side of the nostril than that due to the triangular cartilage. But the most frequent and troublesome form of obstruction at the nostril is the result of an underdeveloped condition of the alæ nasi. This is itself due to nasal obstruction further back, and most usually to adenoids in early life. The nostril is unduly narrow, and the dilatator muscles undeveloped, so that they fall in during inspiration.

It is proposed to deal only in outline with the general principles involved in the treatment of the conditions described. It is first necessary to exclude the cases in which obstruction is due to recent catarrh or to inflammation kept up by disease of the accessory sinuses, and so forth. The treatment of adenoids has lately been discussed in these columns, and need not detain us now; it is perhaps advisable to repeat that removal of the adenoid is not always sufficient except in early childhood, and that the simultaneous treatment of intranasal causes of obstruction is often necessary. It must never be forgotten, when treating intranasal obstruction, that the turbinal bodies subserve the important function of filtering and moistening the inspired air, and that, if they are too freely removed, crusting within the nose, dryness of the throat, and many of the ill-effects of mouth-breathing may follow. Therefore, if removal of turbinals is necessary, neither too much nor too little must be taken away, and considerable experience and judgment is required. It is very difficult to avoid removing too much with the spokeshave, and this instrument should very rarely be used; it is better as a general rule to remove the anterior and posterior ends and to leave the middle part of the turbinal body. The soft posterior end is best removed by means of a snare passed down the nose and hooked over the end with the finger introduced into the naso-pharynx; if it is important to avoid a general anæsthetic an attempt may be made to engage the end in the snare without the aid of the finger by giving the loop a kink so that it tends, on closing, to bend toward the side required. The anterior end of the turbinal is excised by cutting through its attachment as far as necessary and introducing a snare into the cut. Cases with soft vascular engorgement, which shrink readily under cocaine, may be treated with the electric cautery, the object being to tie down the erectile tissue by means of scars. This treatment does not interfere with occupation, and can be made quite painless, but it is only suitable to cases of soft vascular swelling, and the effects are not permanent, but tend to cease after a period of one to two years. When the septum is deflected it is bad practice to attempt to give relief by removing turbinal tissue, and in most cases of nasal obstruction it is best to operate on the septum whenever possible. Especially in the difficult cases of general narrowness of the nasal passages submucous resection of the septum, which reduces the thickness of this part to a minimum, usually gives the best results. In the rare cases, in which the deflection extends very far back in the vomer, it may be advisable to divide the septum after Moure's method and keep it in position for a fortnight with a rubber splint, but there is always a risk of some recurrence. Spurs are usually part of a general deflection, and are less often treated separately since the introduction of submucous resection, but occasionally they may be sawn off with advantage after raising a flap of muco-periosteum. Narrowness and collapse of the alæ are most difficult to treat; any obstruction within the nares should be remedied, more especially any septal deviation near the alar groove. Various patterns of dilator are made to be worn in the nostril, but are usually found to be too uncomfortable. The defect is due to neglected obstruction, and early treatment of this, with systematic breathing exercises, offers the best hope of prevention and cure.

